# Feasibility of a Mind-Body Program for Chronic Pain: A Randomized Clinical Trial

**DOI:** 10.1001/jamanetworkopen.2025.15685

**Published:** 2025-06-16

**Authors:** Jonathan Greenberg, Julia E. Hooker, Katherine A. McDermott, Danielle E. La Camera, Julie R. Brewer, Claire L. Szapary, Tamara J. Somers, Francis Keefe, Sarah A. Kelleher, Hannah M. Fisher, John Burns, Rebecca Jeddi, Ronald Kulich, Gary I. Polykoff, Robert A. Parker, Allison Diachina, Sara Hogan, Natalie Chou, Latrice Yates, Ana-Maria Vranceanu

**Affiliations:** 1 Center for Health Outcomes and Interdisciplinary Research, Department of Psychiatry, Massachusetts General Hospital, Boston; 2 Department of Psychiatry, Harvard Medical School, Boston, Massachusetts; 3 Department of Psychiatry and Behavioral Sciences, Duke University School of Medicine, Durham, North Carolina; 4 Department of Psychiatry and Behavioral Sciences, Rush University, Rush University Medical Center, Chicago, Illinois; 5 Department of Family and Preventive Medicine, Rush University, Rush University Medical Center, Chicago, Illinois; 6 Department of Anesthesia, Critical Care and Pain Medicine, Massachusetts General Hospital, Boston; 7 Department of Physical Medicine and Rehabilitation, Massachusetts General Hospital, Boston; 8 Biostatistics Center, Massachusetts General Hospital, Boston; 9 Duke University School of Medicine, Durham, North Carolina; 10 Rush University Medical Center, Chicago, Illinois

## Abstract

**Importance:**

Increasing physical function for individuals in chronic pain is challenging. Despite the Initiative on Methods, Measurement, and Pain Assessment in Clinical Trials guidelines, no pain trial has comprehensively assessed multimodal physical function (ie, self-reported, performance-based, and objective or step count–based measures), and most of these trials have limited racial and ethnic diversity.

**Objective:**

To test the feasibility of a mind-body walking program and a health education program among geographically, racially, and ethnically diverse sedentary adults with chronic pain.

**Design, Setting, and Participants:**

This single-blind, 2-arm, feasibility randomized clinical trial was conducted at Massachusetts General Hospital in the Northeast, Duke University in the Southeast, and Rush University in the Midwest, capturing a racially, ethnically, and geographically diverse US population. Recruitment occurred between April 2023 and January 2024. Participants were sedentary adults with chronic musculoskeletal pain. After baseline assessment, participants were randomized to either the mind-body walking program or the health education program. Given the pilot nature of this trial, all analyses were conducted on the observed data rather than following intent-to-treat principles.

**Interventions:**

Both interventions consisted of 10 weekly hour-long, in-person group sessions.

**Main Outcomes and Measures:**

Primary outcomes were feasibility benchmarks, including feasibility of recruitment, treatment arms, assessments, participant retention, racial and ethnic diversity attainment, treatment expectancy, treatment credibility, participant satisfaction, and treatment fidelity.

**Results:**

Ninety-two participants were randomized to the mind-body walking program (n = 47) or the health education program (n = 45). Participants had a mean (SD) age of 57 (14.3) years; were predominantly females (69 [75.0%]); and included Asian (2.2%), Black or African American (44.6%), Hispanic (5.4%), non-Hispanic (83.7%), and White (40.2%) individuals. Both mind-body walking and health education interventions met the benchmarks for feasibility of treatment arms (95.7% [44 of 46 patients] and 82.2% [37 of 45 patients]), treatment credibility (100% (41 of 41 patients] and 87.2% [34 of 39 patients]), treatment fidelity (9.93 and 9.95 scores), assessment (93.0% accuracy), and participant retention (93.6% [44 of 47 patients] and 86.7% [39 of 45 patients]). Benchmarks for treatment expectancy (85.7% [36 of 42 patients] vs 66.7% [26 of 39 patients]) and participant satisfaction (97.6% [41 of 42 patients] vs 75.0% [30 of 40 patients]) were met in the mind-body walking program but not in the health education program. The racial and ethnic diversity benchmark was met (54.3% [50 of 92] vs ≥38.0% planned). The recruitment benchmark was not met (69.7% [92 of 132] of eligible participants recruited vs ≥80.0% planned).

**Conclusions and Relevance:**

In this trial, both a mind-body walking program and a health education program were feasible at 3 geographically, racially, and ethnically diverse academic medical centers. The findings support and inform a fully powered, multisite, future efficacy trial of these interventions.

**Trial Registration:**

ClinicalTrials.gov Identifier: NCT05700383

## Key Points

**Question:**

Do a 10-week mind-body walking program and a health education program meet a priori–set feasibility benchmarks among adults with chronic pain across 3 academic medical centers?

**Findings:**

In this feasibility randomized clinical trial involving 92 geographically, racially, and ethnically diverse participants, both interventions were found to be feasible and acceptable. The mind-body walking program exceeded all treatment-specific benchmarks, while the health education program exceeded many benchmarks for feasibility.

**Meaning:**

The results support a future multisite efficacy trial of the mind-body walking program vs the health education program.

## Teaser

This randomized clinical trial evaluates the feasibility of a mind-body walking program and a health education program among a geographically and racially and ethnically diverse sample of sedentary adults with chronic pain.

## Introduction

Chronic pain (ie, pain persisting >3 months^1,2^) is prevalent, with higher incidence rates than diabetes, depression, and hypertension.^3^ Chronic pain is challenging to treat and is associated with impairments to physical function,^4,5^ considerably affecting individuals’ ability to engage in everyday activities. Walking is a safe and accessible behavioral target to help improve physical function among people with chronic pain.^6,7^ However, fear of increased pain as a result of movements such as walking (kinesiophobia) and catastrophic thinking about pain are common and represent key barriers to increasing walking and improving physical function in this population.^8,9^ Mind-body interventions can improve kinesiophobia, pain catastrophizing, and adaptive coping with pain.^10,11,12^ They demonstrate promise in reducing barriers to activity and may offer an effective avenue for increasing physical function.^12,13^

Physical function is not a uniform construct, and some aspects of physical function are difficult to increase among individuals with chronic pain.^14,15^ The Initiative on Methods, Measurement, and Pain Assessment in Clinical Trials (IMMPACT) recommends comprehensive assessment of physical function (eg, self-reported, performance-based, and objective or step count–based) in pain clinical trials.^14^ However, measurement of all 3 types of physical function remains extraordinarily scarce in pain trials.^16^ To help improve multimodal physical function among individuals with chronic pain, we iteratively developed and refined a mind-body walking program.^12,13^ This mind-body program targets increased walking and uses step tracking to provide real-time feedback and reinforcement of walking goals.^12,13^ In a feasibility trial, we found the mind-body walking program to be feasible, acceptable, and associated with self-reported and performance-based physical function^11,12^ and to show improvement in potential mechanisms.^10,17^ Improvements in step count were limited to sedentary individuals^12^ (ie, those with <5000 daily steps on average^18^). The feasibility trial was conducted at a single site and limited by a lack of racial and ethnic diversity. Given the disproportionate impact of chronic pain on racial and ethnic minority individuals and prominent health disparities in the field,^19,20^ testing the mind-body walking intervention in a more racially and ethnically diverse sample is a high priority.

In preparation for a fully powered efficacy trial, we optimized the mind-body walking program. In this multisite feasibility randomized clinical trial (RCT), the primary objective was to test the feasibility of a mind-body walking program and a health education program, a comparison intervention, among geographically, racially, and ethnically diverse sedentary adults with chronic pain. We hypothesized that both programs would meet a priori feasibility benchmarks.^21^ Furthermore, as a secondary objective, we explored whether participants in the mind-body walking program showed improvement in putative mechanisms (pain catastrophizing, kinesiophobia, adaptive coping, and mindfulness) and treatment outcomes such as multimodal physical function (eg, self-reported, performance-based, and objective or step count–based), emotional function (eg, anxiety and depression), and pain. We also explored whether the putative mechanisms would correlate with outcomes to support the conceptual model (eFigure in Supplement 1) and test for signals of mechanistic target engagement, consistent with Science of Behavior Change principles.^22^

## Methods

### Design, Setting, and Participants

A complete description of study procedures is provided in the trial protocol (Supplement 1).^21^ We conducted a multisite, single-blind, 2-arm feasibility RCT at 3 academic medical centers in the US: Massachusetts General Hospital in the Northeast, Duke University in the Southeast, and Rush University in the Midwest. These academic medical centers were selected to capture a racially and ethnically and geographically diverse national population. All study procedures were approved by the institutional review boards at Massachusetts General Hospital, Duke University, and Rush University. Participants provided written informed consent. We followed the Consolidated Standards of Reporting Trials (CONSORT) reporting guideline.

Recruitment occurred between April 2023 and January 2024. Participants were recruited via referrals from established clinicians at pain clinics and through medical record review. We used flyers in clinics and the community as well as social media and institutional participant-recruitment websites.

Inclusion criteria were as follows: (1) age 18 years or older; (2) 3 or more months of self-reported chronic musculoskeletal pain; (3) ability to speak English; (4) ability to walk for 6 or more minutes; (5) willingness to participate in a 10-week group-based program; (6) no changes to psychotropic or pain medication dose in the past 6 weeks, and no changes expected for the duration of the study; (7) access to a wireless-capable device; and (8) meeting at least 2 of 3 self-reported sedentary criteria (ie, sitting ≥8 hours per day, exercising <3 days per week for 30 minutes a day, or walking ≥30 minutes per day). Exclusion criteria were as follows: (1) medical condition expected to worsen in the coming 6 months, (2) untreated psychiatric illness, (3) active suicidal ideation with plan or intent, (4) untreated and active substance use disorder, (5) mindfulness and meditation practice more than 3 times per week for more than 45 minutes, (6) regular use of a smartwatch and refusal to use only study-provided devices during participation, (7) inability to walk or using a wheelchair, and (8) mild cognitive decline as determined by 4 or more errors on the Short Portable Mental Status Questionnaire for those aged 65 years or older.

### Procedures

Participants completed baseline assessments 1 week prior to the first intervention session. The baseline assessment consisted of informed consent, self-reported surveys, the 6-Minute Walk Test (6MWT), and distribution of an accelerometer (ActiGraph wGT3X-BT; ActiGraph LLC) to wear for 1 week. All assessments were repeated 1 week after the intervention. Participants completed assessments on the secure Research Electronic Data Capture (Vanderbilt University) platform, with a research assistant (including D.E.L., J.R.B., A.D., N.C., and L.Y.) present to answer questions. Participants received up to $945 for completing all assessments, procedures, and sessions.

### Treatment Conditions

At the end of the baseline assessment, participants were randomized to either the mind-body walking program or the health education program on a 1:1 ratio (Figure). We used block randomization stratified by site and programmed by the biostatistician (R.A.P.). Both conditions consisted of 60-minute sessions in groups of 3 to 8 participants delivered in person for 10 weeks by a doctoral-level psychologist (including J.E.H., K.A.M., H.M.F., and R.J.). The eTable in Supplement 2 outlines session content for both interventions. Intervention content was previously described in detail.^21^

**Figure.**
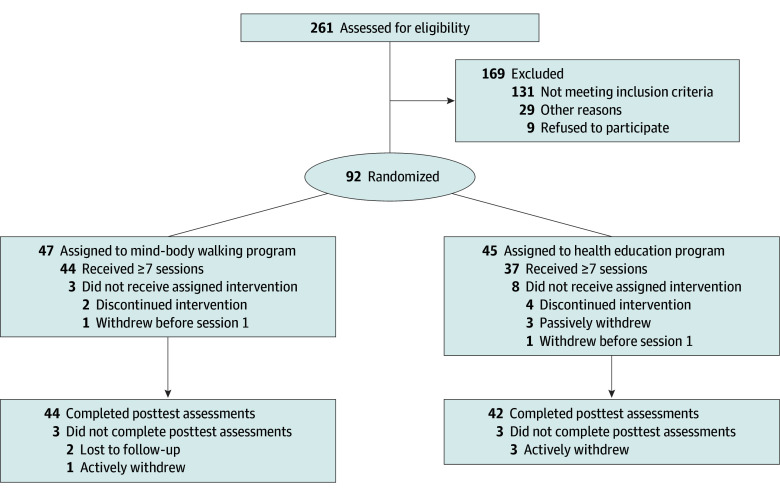
Participant Flow Flowchart of participants’ disposition throughout the study.

#### Mind-Body Walking Program

The mind-body walking program (GetActive-Fitbit) is a skills-based intervention. Its foundational skill is quota-based pacing, consisting of gradual increases in activity based on step-count goals that are noncontingent on pain.^12,23^ To aid quota-based pacing, participants received a step-tracking device (Fitbit Inspire 3; Google) and weekly individualized step goals based on their step-count data. Consistent with prior research,^12,23^ step goals were increased by 10% each week if participants reached the prior week’s goal or were maintained if they did not reach the goal. For participants who missed a step goal for 2 weeks in a row, the new goal was set to 10% more than their observed step count for that week.

Session content included mind-body skills (eg, deep breathing, body scan, and self-compassion) to reduce reactivity to pain and increase activity engagement; pain behavior awareness skills to understand the downward spiral of negative thoughts, feelings, and inactivity that perpetuates pain interference; and cognitive skills to identify unhelpful thoughts and encourage adaptive thinking. Participants completed home practice of skills and logged practice daily via text message–based surveys.

#### Health Education Program

The health education program (Healthy Living for Pain) is a comparison education control intervention (adapted from the Stony Brook Health Enhancement Program^24^) consisting of lifestyle educational content, including nutrition, sleep, exercise, and navigating the health care system. Session length and social support from both clinicians and fellow participants were similar to those in the mind-body walking program. Participants also engaged in home practice (journaling), with daily text message reminders.

### Study Assessments

#### Demographic and Clinical Factors

Table 1 details demographic and clinical factors. These data were self-reported by participants.

**Table 1.** Descriptive Statistics for All Participants

Characteristic	Participants, No. (%)		
	Total (n = 92)	Mind-body walking program (n = 47)	Health education program (n = 45)
Age, mean (SD), y	57.5 (14.3)	55.1 (13.6)	60.1 (14.8)
Sex			
Male	23 (25.0)	10 (21.3)	13 (28.9)
Female	69 (75.0)	37 (78.7)	32 (71.1)
Race^a^			
Asian	2 (2.2)	1 (2.1)	1 (2.2)
Black or African American	41 (44.6)	21 (44.7)	20 (44.4)
White	37 (40.2)	17 (36.2)	20 (44.4)
More than 1 race	8 (8.7)	5 (10.6)	3 (6.7)
Did not answer	4 (4.3)	3 (6.4)	1 (2.2)
Ethnicity^a^			
Hispanic	5 (5.4)	3 (6.4)	2 (4.4)
Non-Hispanic	77 (83.7)	39 (83.0)	38 (84.4)
Did not answer	10 (10.9)	5 (10.6)	5 (11.1)
Educational level			
<High school	2 (2.2)	1 (2.1)	1 (2.2)
High school diploma or GED	18 (19.6)	12 (25.5)	6 (13.3)
Some college or associate’s degree	28 (30.4)	11 (23.4)	17 (37.8)
Bachelor’s degree	25 (27.2)	15 (31.9)	10 (22.2)
Graduate or professional degree	15 (16.3)	6 (12.8)	9 (20.0)
Did not answer	4 (4.3)	2 (4.3)	2 (4.4)
Employment status			
Full-time	21 (22.8)	9 (19.1)	12 (26.7)
Part-time	7 (7.6)	4 (8.5)	3 (6.7)
Keeping house/housemaker	5 (5.4)	4 (8.5)	1 (2.2)
Other^b^	9 (9.8)	4 (8.5)	5 (11.1)
Retired	30 (32.6)	14 (29.8)	16 (35.6)
Unemployed	13 (14.1)	8 (17.0)	5 (11.1)
Did not answer	7 (7.6)	4 (8.5)	3 (6.7)
Household income, $			
<10 000	9 (9.8)	6 (12.8)	3 (6.7)
10 000 to <15 000	10 (10.9)	5 (10.6)	5 (11.1)
15 000 to <20 000	2 (2.2)	1 (2.1)	1 (2.2)
20 000 to <25 000	6 (6.5)	3 (6.4)	3 (6.7)
25 000 to <35 000	8 (8.7)	3 (6.4)	5 (11.1)
35 000 to <50 000	12 (13.0)	9 (19.1)	3 (6.7)
50 000 to <75 000	7 (7.6)	4 (8.5)	3 (6.7)
≥75 000	20 (21.7)	9 (19.1)	11 (24.4)
Did not answer	18 (19.6)	7 (14.9)	11 (24.4)
Marital status			
Single, never married	29 (31.5)	16 (34.0)	13 (28.9)
Married	30 (32.6)	16 (34.0)	14 (31.1)
Living with significant other	3 (3.3)	1 (2.1)	2 (4.4)
Separated or divorced	16 (17.4)	9 (19.1)	7 (15.6)
Widowed	9 (9.8)	2 (4.3)	7 (15.6)
Did not answer	5 (5.4)	3 (6.4)	2 (4.4)
Pain location			
Back, neck, or chest	59 (64.1)	27 (57.4)	32 (68.1)
Head or face	14 (15.2)	6 (12.8)	8 (17.8)
Upper extremity	32 (34.8)	18 (38.4)	14 (29.8)
Lower extremity	60 (65.2)	26 (55.3)	34 (72.3)
Widespread or nonspecific	20 (21.7)	8 (17.0)	12 (25.5)
Unclear	5 (5.4)	4 (8.5)	1 (2.1)
Pain location			
Single	15 (16.3)	10 (21.3)	5 (10.6)
Multiple	77 (83.7)	37 (78.7)	40 (88.9)

Abbreviation: GED, General Educational Development.

^a^ Race and ethnicity were self-reported by participants.

^b^ Other employment included disability or Supplemental Security Income and no answer or unknown.

#### Feasibility Benchmarks

This trial’s primary goal was to test feasibility benchmarks based on prior and similar intervention feasibility studies.^12,13,25,26^ Benchmarks included feasibility of recruitment, treatment arms, assessments, participant retention, racial and ethnic diversity attainment, treatment expectancy, treatment credibility, participant satisfaction, and treatment fidelity. Table 2 provides benchmark definitions.

**Table 2.** Feasibility Benchmarks

Marker	Proposed benchmark	Observed benchmark
Recruitment: Assessed by the number of eligible patients who agree to participate	≥80% Of eligible sedentary patients with chronic pain enrolling after screening	69.7% Of eligible patients (92 of 132) agreed to participate
Racial and ethnic diversity attainment: Assessed by the number of racial and ethnic minority patients who agree to participate	≥38% Of participants being from racial and ethnic minority groups	54.3% Of participants (50 of 92) were from racial and ethnic minority groups
Treatment arms: Assessed by the number of participants who attend 70% of sessions	≥80% Of patients with chronic pain attending ≥7 of 10 sessions (70%) for each program	Mind-body walking program: 95.7% (44 of 46 patients) Health education program: 82.2% (37 of 45 patients)
Treatment credibility: Assessed with the Credibility/Expectancy Questionnaire	≥80% Of patients with chronic pain having credibility scores higher than the questionnaire’s midpoint score	Mind-body walking program: 100% (41 of 41 patients) Health education program: 87.2% (34 of 39 patients)
Treatment expectancy: Assessed with the Credibility/Expectancy Questionnaire	≥80% Of patients with chronic pain having expectancy scores higher than the questionnaire’s midpoint score	Mind-body walking program: 85.7% (36 of 42 patients) Health education program: 66.7% (26 of 39 patients)
Treatment fidelity: Assessed with fidelity checks of 20% of group sessions	≥8 Score (by study therapists) on adherence and competency of the mind-body walking program and health education program group sessions, with 1 indicating poor and 10 indicating outstanding	Mind-body walking program: 9.93 score Health education program: 9.95 score
Participant satisfaction: Assessed with the Client Satisfaction Questionnaire	≥80% Of patients with chronic pain having satisfaction scores higher than the questionnaire’s midpoint score	Mind-body walking program: 97.6% (41 of 42 patients) Health education program: 75.0% (30 of 40 patients)
Participant retention (postintervention): Percentage of participants with completed self-reported, performance-based, and objective or step count–based data	≥80% Of patients with chronic pain having valid data	Mind-body walking program: 93.6% (44 of 47 patients) Health education program: 86.7% (39 of 45 patients)
Assessments: Percentage accuracy of standardized protocol checklists for all assessments	≥90% Accuracy of standardized protocol checklists for all assessments	93.0% Accuracy achieved

For treatment fidelity, trained clinician raters who were not involved with the intervention rated 20% of sessions across each site. Fidelity was rated for session-specific elements and nonspecific factors (eg, time management).

The Credibility/Expectancy Questionnaire^27^ has two 3-item subscales to assess treatment credibility and expectation about benefits gained from the intervention. Score range for the credibility and expectancy subscales is 3 to 27 after item standardization, with the highest score indicating high credibility and high expectancy. The Client Satisfaction Questionnaire^28^ assessed participant satisfaction with the intervention. Score range for the 3-item Client Satisfaction Questionnaire is 3 to 12, with the highest score indicating high satisfaction. These measures were administered after the intervention.

### Treatment Outcomes

#### Multimodal Physical Function

We assessed step count–based physical function using an accelerometer (ActiGraph wGT3X-BT; ActiGraph LLC), which participants wore on their nondominant hand for 1 week prior to the start of group sessions. We tracked participants’ activity via an application (Centrepoint Sync; ActiGraph LLC). Participants returned the device at the first group session if they completed 4 or more days of valid wear data (>10 waking hours per day), or participants were asked to wear the device until they reached the requirement.

Additional physical function assessments included the 6MWT^29^ in which participants were instructed to walk (including with assistive devices) in the clinic for 6 minutes at a fast pace. The 6MWT scores depict the number of meters walked in 6 minutes, with higher scores indicating higher performance-based physical function. Self-reported physical function was assessed using the World Health Organization Disability Assessment Scale 2.0 (WHODAS; score range: 0-100, with the highest score indicating low physical function).^30^

#### Pain, Emotional Function, and Perceived Improvement

The Pain, Enjoyment of Life, and General Activity Scale^31^ uses items drawn from the Brief Pain Inventory^32^ to assess pain intensity and interference in the past week. The score range is 0 to 10, with the highest score indicating high pain intensity and interference.

Emotional functioning was measured using the total score from the Patient-Reported Outcomes Measurement Information System (PROMIS). The PROMIS Short Form v1.0 Depression 8b scale assesses negative mood, engagement in daily living, and social components.^33^ The PROMIS Short Form v1.0 Anxiety 8a scale assesses fear, worry, hyperarousal, and somatic symptoms.^34^ These PROMIS Depression and Anxiety scales yield a mean (SD) standardized *t* score of 50 (10), with higher scores indicating higher symptoms of depression or anxiety.

The Patient Global Impressions of Change instrument was used to assess perceived improvements due to the intervention (eg, in pain management, physical function, and emotional function).^35^ Score range is 1 to 7, with the highest score indicating “very much improved.”

### Putative Mechanisms

#### Pain Catastrophizing and Kinesiophobia

The Pain Catastrophizing Scale^36^ captured magnification, helplessness, and rumination about pain. Its scores range from 0 to 52, with the highest score indicating high catastrophizing. The Tampa Kinesiophobia Scale^37^ assessed fear of pain and injury due to movement. Scores range from 17 to 64, with the highest score indicating high kinesiophobia.

#### Adaptive Coping and Mindfulness

The Measure of Current Status (MOCS-A) assessed the ability to engage in general coping skills (eg, relaxation, confidence in coping abilities, and assertiveness).^38^ We used the total score analysis (score range: 0-52, with the highest score indicating high levels of adaptive coping). The Cognitive and Affective Mindfulness Scale–Revised measured attention regulation, present focus, and nonjudgmental awareness.^39^ It has a score range of 12 to 48, with the highest score indicating high self-reported mindfulness.

### Statistical Analysis

The power analysis for feasibility benchmarks was based on enrolling 90 participants, granting an 80% probability of meeting all feasibility benchmarks if the expected proportion of participants enrolling, completing 7 or more of 10 sessions, rating the program as credible and satisfactory, and completing posttreatment follow-up was at least 89% and if the expected proportion of protocol checklists deemed accurate was at least 94%.

A priori–set feasibility benchmarks were calculated using frequencies and proportions (Table 2). Given the lack of power to reliably detect group differences, and consistent with guidelines for pilot trials,^40,41^ we refrained from conducting between-group comparisons. Rather, we calculated within-group changes in outcomes and putative mechanisms via within-group paired *t* tests and corresponding effect sizes via Cohen *d*. To gauge preliminary mechanistic target engagement, we performed bivariable correlations between baseline-to-postintervention changes in intervention outcomes and changes in the putative mechanisms, consistent with the conceptual model (eFigure in Supplement 2).

Given the pilot nature of this trial, all analyses were conducted on the observed data rather than following intent-to-treat principles. All analyses were conducted in SPSS version 27 (IBM) and Microsoft Excel (Microsoft Corp). Two-sided *P* < .05 indicated statistical significance.

## Results

We enrolled 92 participants. We obtained baseline and postintervention data from 44 of 47 participants (93.6%) in the mind-body walking program and 39 of 45 participants (86.7%) in the health education program. Participants had a mean (SD) age of 57 (14.3) years; included 69 females (75.0%) and 23 males (25.0%); and 2 identified as Asian (2.2%), 41 as Black or African American (44.6%), 5 as Hispanic (5.4%), 77 as non-Hispanic (83.7%), and 37 as White (40.2%) individuals, with 8 (8.7%) reporting more than 1 race and 14 (15.2%) choosing not to answer. Table 1 shows self-reported patient demographic and clinical characteristics.

### Feasibility Benchmarks

Table 2 details feasibility benchmarks for both interventions. The mind-body walking program exceeded all treatment-specific benchmarks, including feasibility of treatment arms (95.7% [44 of 46 patients]), treatment credibility (100% [41 of 41 patients]), treatment expectancy (85.7% [36 of 42 patients]), treatment fidelity (9.93 score), participant retention (93.6% [44 of 47 patients]), and participant satisfaction (97.6% [41 of 42 patients]). The health education program exceeded the benchmarks for feasibility of treatment arms (82.2% [37 of 45 patients]), treatment credibility (87.2% [34 of 39 patients]), treatment fidelity (9.95 scores), and participant retention (86.7% [39 of 45 patients]) but not for treatment expectancy (66.7% [26 of 39 patients]) or participant satisfaction (75.0% [30 of 40 patients]).

The benchmark for feasibility of recruitment was not met: 69.7% (92 of 132) of eligible participants were recruited, but the benchmark was 80.0% or greater. The benchmark for feasibility of racial and ethnic diversity was met, with 54.3% (50 of 92) of participants identifying as racial or ethnic minority individuals (benchmark = ≥38%). The benchmark for feasibility of assessments (≥90%) was also met with 93% accuracy of standardized protocol checklists for all assessments.

### Exploratory Change in Intervention Outcomes

Table 3 presents means (SDs) for all measures in both groups at baseline and after intervention. Overall, physical function levels on all measures were numerically lower than those in the single-site RCT,^12^ possibly due to the new inclusion criterion for sedentariness.

**Table 3.** Outcome Measures

Measure	Mean score (SD)		Mean pre- to postintervention change (95% CI)	*t* Test	*P* value	Cohen *d*
	Baseline	Postintervention				
**Mind-body walking program**						
Step counts, No.^a^	2641.43 (1735.57)	3694.77 (2175.43)	1101.05 (672.72 to 1529.37)	5.18	<.001	0.78
6MWT distance, m^b^	289.62 (77.61)	339.20 (80.32)	48.60 (34.43 to 62.87)	6.87	<.001	1.04
Anxiety^c^	57.25 (10.49)	55.04 (9.84)	−1.22 (−3.32 to 0.87)	−1.18	.25	0.18
Depression^d^	54.67 (9.48)	52.81 (8.53)	−1.07 (−3.23 to 1.10)	-.99	.33	0.15
Physical function^e^	48.79 (22.35)	35.21 (19.09)	−14.58 (−18.75 to −10.41)	−7.07	<.001	1.13
Pain intensity and interference^f^	6.36 (2.05)	5.05 (2.41)	−1.29 (−2.05 to −0.52)	−3.39	.002	0.52
Pain catastrophizing^g^	20.47 (11.30)	11.70 (9.60)	−8.11 (−10.60 to −5.63)	−6.58	<.001	0.99
Kinesiophobia^h^	39.98 (5.39)	34.88 (6.86)	−4.81 (−6.98 to −2.64)	−4.47	<.001	0.67
Adaptive coping^i^	2.27 (0.60)	2.50 (0.68)	0.18 (0.02 to 0.35)	2.29	.03	0.34
Mindfulness^j^	32.11 (5.68)	34.33 (5.97)	1.82 (0.46 to 3.18)	2.69	.01	0.41
Perceived improvement in pain management^k^	NA	6.20 (0.70)	NA	NA	NA	NA
Perceived improvement in physical function^k^	NA	5.93 (0.87)	NA	NA	NA	NA
Perceived improvement in emotional function^k^	NA	6.00 (0.75)	NA	NA	NA	NA
**Health education program**						
Step counts, No.^a^	3177.11 (2573.83)	3032.90 (2668.91)	45.33 (−417.47 to 508.12)	0.20	.84	0.03
6MWT distance, m^b^	260.28 (76.26)	307.37 (80.11)	40.56 (26.75 to 54.38)	5.94	<.001	0.94
Anxiety^c^	56.24 (10.14)	54.69 (9.08)	−1.15 (−3.42 to 1.12)	−1.02	.31	0.16
Depression^d^	54.00 (9.54)	52.14 (9.49)	−2.05 (−4.66 to 0.55)	−1.59	.12	0.25
Physical function^e^	44.86 (25.04)	39.24 (26.02)	−2.57 (−7.60 to 2.46)	−1.04	.31	0.18
Pain intensity and interference^f^	6.72 (1.78)	5.89 (2.26)	−0.76 (−1.41 to −0.12)	−2.38	.02	0.37
Pain catastrophizing^g^	19.71 (13.56)	16.77 (13.81)	−2.62 (−5.49 to 0.26)	−1.84	.07	0.28
Kinesiophobia^h^	40.37 (5.84)	40.13 (5.70)	−0.39 (−2.27 to 1.49)	−0.42	.68	0.07
Adaptive coping^i^	2.27 (0.84)	2.37 (0.79)	0.07 (−0.12 to 0.25)	0.71	.48	0.11
Mindfulness^j^	34.43 (7.98)	34.32 (7.52)	−0.43 (−1.90 to 1.04)	−0.59	.56	0.09
Perceived improvement in pain management^k^	NA	5.38 (1.23)	NA	NA	NA	NA
Perceived improvement in physical function^k^	NA	5.15 (1.29)	NA	NA	NA	NA
Perceived improvement in emotional function^k^	NA	5.40 (1.32)	NA	NA	NA	NA

Abbreviations: 6MWT, 6-Minute Walk Test; NA, not applicable.

^a^ Measured with ActiGraph wGT3X-BT accelerometer (ActiGraph LLC).

^b^ Measured with 6MWT; scores depict the number of meters walked in 6 minutes, with higher scores indicating higher performance-based physical function.

^c^ Measured with Patient-Reported Outcomes Measurement Information System (PROMIS) Short Form v1.0 Anxiety 8a; higher scores indicate higher symptoms of anxiety.

^d^ Measured with PROMIS Short Form v1.0 Depression 8b; higher scores indicate higher symptoms of depression.

^e^ Measured with World Health Organization Disability Assessment Schedule; score range: 0-100, with the highest score indicating low physical function.

^f^ Measured with Pain, Enjoyment of Life, and General Activity Scale; score range: 0-10, with the highest score indicating high pain intensity and interference.

^g^ Measured with Pain Catastrophizing Scale; score range: 0-52, with the highest score indicating high catastrophizing.

^h^ Measured with Tampa Scale of Kinesiophobia Scale; score range: 17-64, with the highest score indicating high kinesiophobia.

^i^ Measured with Measure of Current Status; score range: 0-52, with the highest score indicating high levels of adaptive coping.

^j^ Measured with Cognitive and Affective Mindfulness Scale-Revised; score range: 12-48, with the highest score indicating high self-reported mindfulness.

^k^ Measured with Patient Global Impressions of Change; score range: 1-7, with the highest score indicating “very much improved.”

Participants in the mind-body walking program exhibited significant improvements of large effect sizes in physical function measured by the 6MWT (Cohen *d* = 1.04; *P* < .001), WHODAS (Cohen *d* = 1.13; *P* < .001), and step count (Cohen *d* = 0.78; *P* < .001) as well as pain catastrophizing (Cohen *d* = 0.99; *P* < .001). Participants exhibited significant improvements of medium effect sizes in pain intensity and interference (Cohen *d* = 0.52; *P* = .002) and kinesiophobia (Cohen *d* = 0.67; *P* < .001). Significant improvements of small effect sizes were found in adaptive coping (Cohen *d* = 0.34; *P* = .03) and mindfulness (Cohen *d* = 0.41; *P* = .01). Perceived improvements in pain management (postintervention mean [SD] score, 6.20 [0.70]), physical function (postintervention mean [SD] score, 5.93 [0.87]), and emotional function (postintervention mean [SD] score, 6.00 [0.75]) were high.

Participants in the health education program exhibited significant improvement of a large effect size in the 6MWT (Cohen *d* = 0.94; *P* < .001) and of small-medium effect size in pain intensity and interference (Cohen *d* = 0.37; *P* = .02). No other effects reached statistical significance. Perceived improvements in pain management (postintervention mean [SD] score, 5.38 [1.23]), physical function (postintervention mean [SD] score, 5.15 [1.29]), and emotional function (postintervention mean [SD] score, 5.40 [1.32]) were moderately high (Table 3).

### Correlations Between Outcomes and Putative Mechanisms

Table 4 presents correlations between outcomes and putative mechanisms following completion of the mind-body walking program. Improvements in self-reported physical function (WHODAS) were correlated with improvements in pain catastrophizing (*r* = 0.67; 95% CI, 0.44-0.81; *P* < .001), kinesiophobia (*r* = 0.39; 95% CI, 0.85-0.63; *P* = .01), and mindfulness (*r* = –0.46; 95% CI, −0.67 to −0.16; *P* = .003). Improvements in depression and anxiety were both associated with improvements in pain catastrophizing (*r* = 0.37 [95% CI, 0.08-0.60; *P* = .01] and *r* = 0.42 [95% CI, 0.14-0.64; *P* = .004]) and mindfulness (*r* = −0.38 [95% CI, −0.60 to −0.08; *P* = .01] and *r* = −0.32 [95% CI, −0.56 to −0.02; *P* = .04]). Improvements in pain were associated with improvements in kinesiophobia (*r* = 0.50; 95% CI, 0.23-0.69; *P* < .001). No other correlations between outcomes and putative mechanisms reached statistical significance.

**Table 4.** Correlations Between Change in Outcomes and Changes in Putative Mechanisms in Mind-Body Walking Program

Measure	*r* (95% CI)				
	Physical function, WHODAS	Walking, 6MWT	Step count, ActiGraph^a^	Depression, PROMIS^b^	Anxiety, PROMIS^c^
Pain catastrophizing, PCS	0.67 (0.44 to 0.81)^d^	−0.07 (−0.36 to 0.23)	−0.09 (−0.38 to 0.21)	0.37 (0.08 to 0.60)^e^	0.42 (0.14 to 0.64)^f^
Kinesiophobia, TSK	0.39 (0.85 to 0.63)^e^	<.001 (−0.30 to 0.30)	−0.15 (−0.42 to 0.16)	0.20 (−0.11 to 0.47)	0.29 (−0.02 to 0.54)
Adaptive coping, MOCS	−0.14 (−0.44 to 0.18)	0.14 (−0.16 to 0.42)	−0.06 (−0.35 to 0.24)	−0.04 (−0.34 to 0.26)	−0.05 (−0.34 to 0.25)
Mindfulness, CAMS-R	−0.46 (−0.67 to −0.16)^f^	−0.02 (−0.31 to 0.28)	−0.03 (−0.32 to 0.27)	−0.38 (−0.60 to −0.08)^e^	−0.32 (−0.56 to −0.02)^e^

Abbreviations: 6MWT, 6-Minute Walk Test; CAMS-R, Cognitive and Affective Mindfulness Scale-Revised; MOCS, Measure of Current Status; PCS, Pain Catastrophizing Scale; PROMIS, Patient-Reported Outcomes Measurement Information System; TSK, Tampa Scale of Kinesiophobia Scale; WHODAS, World Health Organization Disability Assessment Scale.

^a^ ActiGraph wGT3X-BT accelerometer (ActiGraph LLC).

^b^ PROMIS Short Form v1.0 Depression 8b.

^c^ PROMIS Short Form v1.0 Anxiety 8a.

^d^ *P* < .001.

^e^ *P* < .05.

^f^ *P* < .01.

## Discussion

Increasing physical function in chronic pain is challenging, and pain trials do not abide by IMMPACT^14^ recommendations to include multimodal physical function assessment. Furthermore, despite prominent racial and ethnic disparities in chronic pain,^19,20^ most available trials have limited racial and ethnic diversity, highlighting a clear need for more diverse samples in pain trials. We presented the results of a multisite trial of the feasibility of a mind-body walking program for chronic pain targeting multimodal physical function vs a health education program among racially and ethnically diverse patients at 3 US academic medical centers. Secondary aims were to test within-group improvements in outcomes and putative mechanisms and preliminary associations between outcomes and putative mechanisms.

Consistent with our first hypothesis, both the mind-body walking program and the health education program met a priori benchmarks for feasibility of treatment arms, treatment credibility, participant retention, treatment fidelity, and assessments. Furthermore, the benchmark for racial and ethnic diversity was exceeded at 54% (benchmark = ≥38%), which was a limitation of the prior single-site feasibility trial.^12^ The benchmark for feasibility of recruitment was not met (70% of eligible participants recruited; benchmark = ≥80%). This unmet benchmark may be attributed to the lag of several weeks between screening and start of the group to ensure there were enough participants per group, during which some participants were no longer available. Reducing this lag may improve this benchmark. Furthermore, while feasibility benchmarks for treatment expectancy and participant satisfaction were met in the mind-body walking program, they were not met in the health education program. This finding might reflect the control group nature of the health education program, which was not geared toward improving pain outcomes; we are currently in the process of optimizing the health education program. The participant retention benchmark was met. Overall, the results support the feasibility of the 2 programs and will help inform the target sample size and other considerations in preparation for a future fully powered multisite efficacy trial.^42^

Consistent with our second and exploratory hypothesis, participation in the mind-body walking program was associated with improvements in intervention outcomes of physical function (eg, self-reported, performance-based, and objective or step count–based) and pain management as well as in all putative mechanisms (ie, pain catastrophizing, kinesiophobia, mindfulness, and adaptive coping). Discrepant from the single-site RCT of the mind-body walking program, improvements in depression and anxiety had small effect sizes and did not reach statistical significance in this underpowered trial.^12^ In contrast to participants in the mind-body walking program, participants in the health education program showed improvement only in the 6MWT and pain intensity and interference. As would be expected, these findings overall indicate that the health education program does not affect the mind-body walking program’s putative mechanisms or most treatment outcomes (eFigure in Supplement 2). This finding provides full support for the use of the health education program as a comparison condition.

Our third and exploratory hypothesis regarding preliminary mechanistic engagement following the mind-body walking program received partial support. Change in self-reported outcomes (eg, physical function, anxiety, depression, and pain) correlated in varying degrees with putative mechanisms of pain catastrophizing, kinesiophobia, and mindfulness. Change in adaptive coping, as captured by MOCS-A, did not correlate with outcomes. One potential reason for this finding is that, while MOCS-A includes some reported use of relaxation (which is directly taught in the mind-body walking program), it also captures coping skills that are not related to relaxation or other skills taught in the program (eg, assertiveness and coping confidence).^38^ Change in performance-based and objective or step count–based physical function did not correlate with any such putative mechanisms. This finding is consistent with results of prior research, including studies^4,17^ indicating limited or absent correlations between self-reported measures and objective performance-based measures across a variety of constructs. This highlights the multifaceted nature of physical function^4^ and the importance of capturing it comprehensively. Such correlations are preliminary given the underpowered sample and feasibility focus of the trial. Overall, findings of self-reported measures are largely consistent with our conceptual model.

### Strengths and Limitations

The strengths of this study include (1) participation of 3 geographically diverse sites; (2) inclusion of racial and ethnic minority individuals underrepresented in pain interventions; (3) the RCT study design; (4) the active comparison group (health education program), which controls for multiple factors nonspecific to the mind-body walking program (eg, support from the group and therapist, demand characteristics); (5) establishing feasibility markers prior to future efficacy testing, thus maximizing rigor; (6) being among the first mind-body study, to our knowledge, to abide by the IMMPACT recommendations to include self-reported, performance-based, and objective or step count–based physical function^14^; and (7) the iterative intervention adaptation process using mixed methods and a theoretically grounded evidence-based framework.^9,43^

Among the trial limitations, we assessed the feasibility of the treatment credibility and expectancy benchmarks post hoc, after the intervention was complete. Asking participants to retroactively reflect on their experiences prior to the program may reduce accuracy, although capturing such measures after the intervention is not uncommon in pain trials.^44,45^ The relatively high monetary compensation (up to $945) may have affected feasibility measures. Outcomes may have been affected by differences between pain conditions and characteristics. Other factors that we did not assess, such as chronic stress and posttraumatic stress disorder, could also have altered the results. Results are not intended to inform the efficacy or effectiveness of the interventions. Rather, the study intended to establish feasibility in multiple and diverse sites to optimize the interventions and study procedures in preparation for a future efficacy trial. Therefore, exploratory analyses (eg, changes in intervention outcomes and mechanistic target engagement) should be interpreted with caution.

## Conclusions

In this RCT of the feasibility of a mind-body walking program and a health education program, both interventions were feasible at 3 geographically, racially, and ethnically diverse sites. The mind-body walking program shows promise in improving multimodal physical function, emotional function, pain management, and putative mechanisms (eg, pain catastrophizing, kinesiophobia, mindfulness, and adaptive coping). Overall, the findings support and inform a fully powered, multisite, future efficacy trial of the mind-body walking program and the health education program.

## Notes

Supplement 1.
Trial Protocol

Supplement 2. **eTable.** Skills and Content Offered in GetActive-Fitbit and HLP Group Sessions **eFigure.** GetActive-Fitbit Intervention Conceptual Model

Supplement 3.
Data Sharing Statement
