# Changes in the Byline

**DOI:** 10.1001/jamanetworkopen.2025.27096

**Published:** 2025-07-18

In the Original Investigation titled “Feasibility of a Mind-Body Program for Chronic Pain: A Randomized Clinical Trial,”^1^ which was published on June 16, 2025, the Thrive Study Team was removed from the byline, and the names of the team members were added to the byline. This article was corrected.^1^
