# Supplementary material for: Feasibility of a Mind-Body Program for Chronic Pain: A Randomized Clinical Trial
Source: JAMA Netw Open. 2025 Jun 16;8(6):e2515685. doi: 10.1001/jamanetworkopen.2025.15685 (PMC12171935; doi:10.1001/jamanetworkopen.2025.15685)
Supplement: Supplement 1. — Trial Protocol [file jamanetwopen-e2515685-s001.pdf]

Supplemental Online Content

Greenberg J, Hooker JE, McDermott KA, et al; and the THRIVE Study Team. Feasibility of a mind-body program for chronic pain: a randomized clinical trial. *JAMA Netw Open*. 2025;8(6):e2515685. doi:10.1001/jamanetworkopen.2025.15685

eTable. Skills and Content Offered in GetActive-Fitbit and HLP Group Sessions

eFigure. GetActive-Fitbit Intervention Conceptual Model

This supplemental material has been provided by the authors to give readers additional information about their work.

© 2025 Greenberg J et al. *JAMA Network Open*

eTable. ***Skills and content offered in GetActive-Fitbit and HLP group sessions***

Session	*GetActive-Fitbit* Topic	Skills and Content	*Healthy Living for Pain* Topic	Content
1	**Taking Charge of Pain**	Pain alarm, common myths about pain, downward and upward spirals, deep breathing	**Program Overview and Chronic Pain**	Program goals, understanding chronic pain, the impact of stress
2	**Pace Yourself**	Noticing unhelpful thoughts, quota-based pacing	**The Connection Between Chronic Pain and Physical Wellness**	Overview of the relationship between chronic pain and health
3	**Mindfulness**	Mindfulness, body scan	**Sleep and Wellness – Connection with Chronic Pain**	Healthy sleeping strategies, cognitive and physical health
4	**Walk All Over Pain**	Mindful walking, identifying meaningful activities	**Exercise and Wellness – Connection with Chronic Pain**	Physical exercise, maintaining healthy weight
5	**Working with Overly Negative Thoughts**	Identifying and challenging Negative Automatic Thoughts (NATs)	**Nutrition I: The Basics – Connection with Chronic Pain**	Basic nutrition, portion size and calories, understanding food labels
6	**Staying in the Upward Spiral**	Building on your gains, “why do you walk?”, identifying personal hotspots	**Nutrition 2: Healthy Weight and Weight Loss – Connection with Chronic Pain**	Eating healthier meals and snacks, eating out healthy, weight loss and BMI
7	**Mindfulness of Pain**	Self-compassion, mindfulness of pain	**Managing Your Healthcare for Chronic Pain**	Communicating with doctors, medical emergencies
8	**Feeling Connected with Others**	Link social connection and pain	**Navigating Pain Medication with Chronic Pain**	Types of pain medication, chronic pain and medication, incorrect medication use, adherence, substance use and alcohol
9	**Promoting Acceptance**	Discuss social isolation, cultivating acceptance	**Social Isolation, Unhealthy Screen Time, and Chronic Pain**	Chronic pain and social isolation, maintaining a healthy digital balance and screen time use
10	**Staying on Track and Maintaining Your Progress**	Review all skills, create personalized plan	**Review of Healthy Living for Pain**	Overview of program

*Table taken from: Hooker, J. E. *et al.* Improving Multimodal Physical Function in Adults with Heterogeneous Chronic Pain: Protocol for A Multisite Feasibility Randomized Control Trial. *J Pain* **25**, 36 (2024).

© 2025 Greenberg J et al. *JAMA Network Open*

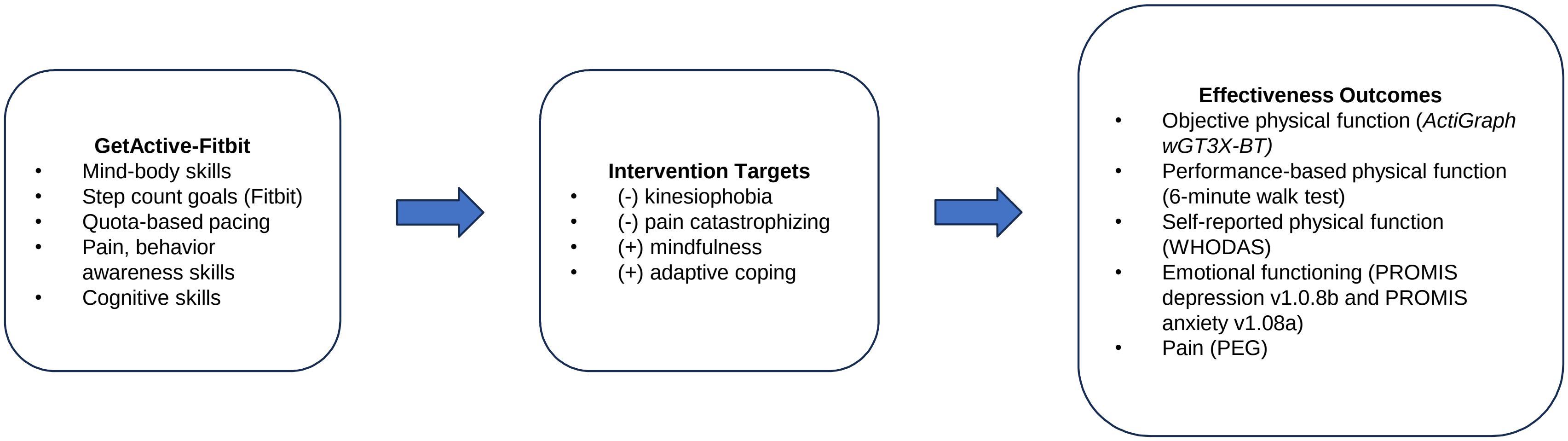

eFigure 1. *GetActive-Fitbit* Intervention Conceptual Model

© 2025 Greenberg J et al. *JAMA Network Open*
