# Supplementary material for: Feasibility of a Mind-Body Program for Chronic Pain: A Randomized Clinical Trial
Source: JAMA Netw Open. 2025 Jun 16;8(6):e2515685. doi: 10.1001/jamanetworkopen.2025.15685 (PMC12171935; doi:10.1001/jamanetworkopen.2025.15685)
Supplement: Supplement 2. — eTable. Skills and Content Offered in GetActive-Fitbit and HLP Group Sessions eFigure. GetActive-Fitbit Intervention Conceptual Model [file jamanetwopen-e2515685-s002.pdf]

Mass General Brigham

Institutional Review Board
Intervention/Interaction Detailed Protocol

Principal Investigator:	Ana-Maria Vranceanu, Ph.D.
Project Title:	Improving multimodal physical function in adults with heterogeneous chronic pain; Multi-site feasibility RCT
Version Date:	3/21/24

1. Background and Significance

Chronic pain is prevalent, costly, and associated with decreased physical function. Chronic pain, defined as persistent pain that lasts more than 3-6 months[1], has an estimated incidence of 100 million adults in the US[2]. The total annual incremental cost of health care due to pain ranges between $560 and $635 billions in the US, which combines the medical costs of pain care and the economic costs related to disability days and lost wages and productivity[2]. As a consequence, NCCIH has designated chronic pain as a national priority area[4]. A large body of research documents that patients with chronic pain have decreased physical function[21,22], regardless of the location and severity of pain[5,23]. Patients with chronic pain also report nonadaptive coping strategies with pain catastrophizing and fear of pain being the most salient, and both associated with decreases in physical function[24–27].

Mind-body programs show promise with chronic pain but fail to meaningfully improve physical function. Over the last decade, psychosocial treatments have evolved toward acceptance of pain and increased function regardless of pain sensations[28–30]. Mind-body programs such as Acceptance and Commitment Therapy and Mindfulness Skills Training focus on engaging in value driven behaviors, even if those are painful[6,30–32]. However, these interventions produce only small to moderate effect sizes for psychosocial outcomes like depression, anxiety, stress, and overall quality of life and effects diminish over time[6,30,33,34]. Further, although IMMPACT [9,35] clearly specifies that physical function should be a required outcome in pain clinical trials, few studies include it[10]. When physical function is included as outcome, effect sizes are small and fade over time[30,36]. <u>There is a critical need for novel interventions to improve physical function, an outcome consistently depicted as top concern by patients with chronic pain[8].</u>

Physical function is a multidimensional construct. Physical function, defined following the ICF[11], implies *"a person's capacity in a set of situations and includes engagement in meaningful aspects of one's life including performing activities of daily living such as household chores, walking, work and self-care"*, is a marker of an individual's health and well-being[22]. IMMPACT recommendations[9] stress the pivotal role of addressing physical function, and urge researchers to: 1) directly target it during pain clinical trials as a primary outcome, and 2) conceptualize it comprehensively not only through self-report measures of activity of daily living but also through performance based measures (functional capacity during a time limited standard test), and objective measures of activity (ambulatory step-count measured by accelerometers that capture daily activity across a specified time period, usually a week[37].) <u>This comprehensive approach to the conceptualization and assessment of physical function aligns with the 2021-2025 NCCIH strategic plan that focuses on whole person health[38].</u> Our recent systematic review[10] showed that **no mind-body clinical trials in chronic pain have comprehensively assessed and addressed improvement in all facets physical function consistent with ICF[11] and IMMPACT[9] criteria.**

Quota based walking is associated with improved outcomes in patients with chronic pain, but it is not incorporated within mind-body programs. This is a missed opportunity. The US Department of Public Health's research has shown a clear relationship between daily walking and several health outcomes such as mortality, cardiovascular disease and cancer[39], with more steps incrementally increasing positive outcomes[40–42]. **Patients with chronic pain are sedentary** (< 5,000 steps a day, which is less than an average healthy adult[43–46]). Deconditioning is common and a significant risk factor for *further* pain conditions and increased disability and overall wellbeing[46] . This contention is consistent with the fear avoidance model[25,47,48] that explains how chronic pain is perpetuated through a disability spiral of decreased activity/deconditioning, negative emotions, and avoidance (Fig. 2) Focus groups with patients with chronic pain have consistently showed that walking is their preferred method of activity[49]. A 2020 Cochrane systematic review[46,50] has showed that walking "is acceptable, beneficial and unlikely to cause harm in people with chronic pain. However, many fear that it would increase their pain in the future" and, as a result, uptake and adherence are problematic[45,46,51] . Quota-based pacing – engagement in an a priori specified activity goal (e.g., quota) that is not contingent on pain – shows promising results[52–54] but decreased mood, pain, coping difficulties (e.g., fear avoidance, catastrophic thinking about pain), programs that are too challenging (e.g., going to the gym), not meaningful, interfering with one's life, or too difficult to implement[50,55–57] have been depicted as important barriers in prior research. **Mind-body skills can bypass these barriers to activity and, if combined with quota-based pacing (walking linked to activities of daily living), may be an effective way to break the disability spiral (fig. 2) and improve all aspects of physical function.**

Fitbits can track and reinforce activity over time while maintaining motivation, and safely allowing individuals to increase step-count. Fitbits are user friendly and low-cost devices that allow individuals to track their step-count and receive real time feedback to increase motivation and reinforce activity. They are also a novel way for clinicians to get objective data on patients' activity, thereby further buttressing motivation, treatment alliance and adherence [13,14,58]. Fitbits can bypass some of the challenges (e.g., problematic adherence, lack of real time feedback) of prior more cumbersome activity tracking devices (e.g., pedometers and accelerometers) [59,60], increasing their ability to act as an intervention[61,62]. **Fitbit**s allow setting step goals that are individualized to each chronic pain patient's ability and gradually increased[16] over time. While the accuracy of measurement of step count with the Fitbit is controversial, and accelerometers are considered the gold standard for assessment of activity in research, Fitbits show higher adherence than accelerometers or pedometers when worn for a long period of time[14,15,63]. Very few studies have examined the Fitbit in patients with chronic pain. Two recent studies used the Fitbit alone (e.g., with no additional skills) to increase activity among people with chronic pain and did not observe significant increases in step-count[64] or self-reported physical function[65]. **Among people with chronic pain, the Fitbit appears promising (good adherence), but does not lead to sustained increase in step count on its own.**

Combining mind-body skills with the Fitbit is a novel solution to the problem of low physical function among people with chronic pain. **Mind-body skills** can bypass barriers to engaging and adhering to activity (e.g., low mood, over-focus on control of pain rather than acceptance, fear avoidance and nonadaptive thoughts about pain), which are also hypothesized mechanisms for improvement in function[66,67]. **Fitbits** can be used to reinforce an individualized quota-based pacing plan with gradual increased activity while enhancing adherence and maintaining motivation. Further, step-count activity goals can be linked to a patient's valued activities[50,55,56], with important implications for sustainability and whole person health.

Conceptual model: We propose that intervention targets such as 1) nonadaptive reactions to nociception (kinesiophobia, and catastrophizing), 2) adaptive coping strategies (e.g., mindfulness, resiliency); 3) social-emotional factors (depression, anxiety, social support, social isolation) will be interrelated and

synergistically lead to improvement in physical function. Activity goal setting skills (pairing of step-count with activities of daily living depicted by patients as important, gradually increasing step goals via quota-based pacing, using the Fitbit live reinforcement of activity) are conceptualized as key factors in improving outcomes in *GetActive-Fitbit*. The program skills act by directly addressing habitual negative affective reactions to pain and negative pain thoughts by focusing on accepting and learning to tolerate pain sensations (including during increased activity[68]), while Fitbit based on operant approaches previously depicted as promising in this population, acts by directly targeting increased activity through "quota-based" pacing and immediate feedback on number of steps achieved[69,70] . In our conceptualization, the mind-body skills are necessary in order to bypass barriers to activity, and the Fitbit is necessary in order to maintain motivation and reinforce activity in real time.

With funding from NCCIH we iteratively developed and refined the first mind-body activity programs (*GetActive and GetActive-Fitbit*) and conducted a single-site, pilot randomized controlled trial in individuals with heterogeneous chronic pain (R34 AT009356; see Figure 4). Both programs showed excellent feasibility, expectancy, and satisfaction in the target population. We also observed, in both programs, within-group improvements in self-report and performance-based physical function across all participants, and in step-count across sedentary participants. Although programs were relatively similar in feasibility markers and signal of improvement in physical function, patients in *GetActive-Fitbit* reported on the Modified Impression of Change that the Fitbit "greatly increased" their ability to improve physical function. Qualitative exit interviews also confirmed preference for using a Fitbit[17]. Program attendance and credibility were superior for *GetActive-Fitbit.* Clinicians noted that it was challenging to assist patients in *GetActive* to gradually increase activity without an objective step goal. Furthermore, although any quantitative analyses must be considered preliminary at this stage, [71,72] we found significant improvement in emotional function in the *GetActive-Fitbit* but not the *GetActive* group. Taken together, these findings support the goal of this 3-year R01 to test multisite feasibility of *GetActive-Fitbit* vs an educational control across geographically diverse sites, which is necessary before conducting a multi-site efficacy trial. A multisite feasibility trial of *GetActive-Fitbit* vs *GetActive* vs control followed by a 3-arm multisite efficacy trial is too expensive and not justified by our preliminary data.

Summary of significance of the proposed NCCIH R01: Chronic pain is a serious public health concern. Improving physical function is a top concern of people with chronic pain[8]. Prior treatments are associated with only small improvement in physical function which tend to fade over time. No prior clinical trials in chronic pain have followed IMMPACT and ICF recommendations of assessing and addressing physical function comprehensively (e.g., self-report, performance based and objective)[10]. **Improving all aspects of physical function is imperative if we are to decrease the medical and economic burden of chronic pain and improve the lives of individuals with chronic pain (e.g., whole person).** We demonstrated that *GetActive-Fitbit* met or exceeded a-priori set feasibility benchmarks and was associated with improvements in comprehensive physical function among sedentary (5,000 steps/day)[18] patients with chronic pain[17]. Before conducting a multisite efficacy trial to demonstrate the efficacy of *GetActive-Fitbit*, we must demonstrate fidelity, feasibility/acceptability benchmarks, and refine our protocols in order to move from a local/single-site to a multi-site randomized controlled trial. Multiple sites allow for strong generalizability, and are necessary to generate a rate of recruitment for a targeted sample that will permit timely enrollment, long-term follow up, and evaluation of treatment mediators and moderators[73]. Further, the multi-site feasibility testing allows us to correct limitations of our prior single-site study including recruitment of racially and ethnically diverse populations, accurate recruitment of the population of interest (chronic pain individuals who take less than 5,000 steps/day) and determining ability to follow-up participants after program completion in order to understand sustainability of improvements. We will also be able to further optimize the protocol and explore systemic barriers (through focus groups with staff) to enhance the rigor and success of the future efficacy testing. This project directly fits the NCCIH priority areas[74] and 2021-2025 strategic plan of: 1) <u>advancing research on the whole person</u> (e.g., we are focused on improving

comprehensive physical function - self-reported, performance based and step-count linked to valued activities, and emotional function); 2) integrating complementary and conventional care (e.g., we are recruiting patients who seek conventional medical care in pain clinics and integrating a mind-body activity within their care); 3) improving health in minorities and eliminating health disparities (e.g., we are specifically focused on recruiting a diverse sample of >38% racial and ethnic minorities); and 4) utilizing complementary and integrative management of pain. This project directly aligns with the NCCIH R01 PAR-21-241 to establish multi-site fidelity and feasibility benchmarks through a non-efficacy RCT. There is an opportunity for a patient centered paradigm shift in how chronic pain is currently treated in pain clinics, with important implications for improving physical function among this high-need population.

2. Specific Aims and Objectives

Aim 1: To test multisite fidelity of training and implementation of *GetActive-Fitbit* vs *HEP*, among adults with heterogeneous, non-malignant, musculoskeletal chronic pain.

Hypothesis: We hypothesize that study clinicians can be trained consistently and competently across sites to achieve implementation of the 2 programs with high adherence and competence based on a-priori set benchmarks.

Aim 2: To evaluate multisite feasibility benchmarks including ability to recruit sedentary chronic pain patients and racial and ethnic minorities for a protocol involving randomization to *GetActive-Fitbit* versus HEP with post-program and 6 months follow up.

Hypothesis: We hypothesize that we will meet a-priori set feasibility benchmarks including ability to recruit and retain racial and ethnic minorities through a 6-months follow-up (38% minorities).

Aim 3: To optimize our fidelity and study implementation protocol procedures in preparation for a future fully powered multisite efficacy RCT through the UH3/UG3 mechanism (PAR-21-243).

Hypothesis: We expect that we will successfully refine training, implementation and procedures using an iterative approach with separate focus groups with patients and staff, and within and cross-site meetings.

3. General Description of Study Design

In **Aim 1**, we aim to test the multisite fidelity of training and implementation of *GetActive-Fitbit* vs *HEP*, internally among study staff. In **Aim 2**, we aim to evaluate multisite feasibility benchmarks for a protocol involving randomization to *GetActive-Fitbit* versus *HEP* with post-program and 6 months follow up. Both programs will be delivered in-person to adults with chronic musculoskeletal pain. In **Aim 3**, we aim to optimize our fidelity and study implementation protocol procedures in preparation for a future fully powered multisite efficacy RCT through the UH3/UG3 mechanism.

During Aim 1, we aimed to test the fidelity of training and implementation of *GetActive-Fitbit* and Health Enhancement Program (HEP). This multisite training included study clinicians at the Massachusetts General Hospital, Duke University, and Rush University sites. The training consisted of presentations reviewing the theoretical and empirical study background, treatment goals, population challenges, process

factors, and study manuals. Following the presentations, training study clinicians role-played key skills and conducted mock groups with staff members. During these mock groups, the training study clinicians followed the study manuals and protocols intended for the single-blind RCT (Aim 2). Fidelity checks are being conducted iteratively to ensure proper training for the implementation of *GetActive-Fitbit* and Health Enhancement Program (HEP). Quantitative assessment of clinicians' knowledge will occur throughout this training process and will mark the final step of their training. This aim of the study will slightly overlap with recruitment for Aim 2 and will be completed by April 15th, 2023, before the first group of participants enroll in the single-blind RCT.

In this current phase of the study (Aim 2), we propose to evaluate the multisite feasibility benchmarks of *GetActive-Fitbit* and *Health Enhancement Program* (*HEP*). This multisite feasibility randomized controlled trial will include Massachusetts General Hospital (MGH), Duke University, and Rush University as study sites. First, adults with chronic pain will be recruited from physician referrals and medical records at Duke and IRB-approved flyers throughout all MGH, Duke, and Rush outpatient clinics and community health centers, and from the community. In the event that a provider refers a patient without sharing contact information and the patient agrees to be contacted by the study team, the research assistant will use the patient's medical record to access this information.

The research assistant will conduct individual phone screens with interested adults to learn more about the study and be screened for eligibility. At Duke, the study team will send a letter via postal mail and/or email and follow up with the potential participant by phone. Participants will also have the option of completing a portion of this screening on their own via REDCap. There will be a QR code and link directing potential participants to the screening REDCap on our study flyers. Eligibility will be determined by the research assistant who conducted the phone screening, and the larger study team, if needed. If eligible, the research assistant will briefly review the consent form with participants who express interest and intent to participate in the study. The research assistant will ask eligible participants to provide dates and times that they are available to attend the group program and maintain a detailed log of this information. Study staff will continue to follow-up with interested participants via phone until a date for the groups has been selected (group times are based off a majority of participants' availability). Once a mutual date and time for the groups has been selected for about 8-20 participants, a research assistant will call eligible participants to schedule an in-person group baseline assessment at each of the 3 sites, which includes the informed consent procedure with a trained research assistant. The research assistant will ask eligible participants who are not available at the set time if they want to be contacted for the next round of groups. Individuals who do not meet study criteria will be offered a resource sheet with relevant health and mental health information.

Eligible participants who are able to attend the scheduled group sessions will meet in person at each of the 3 sites for the baseline assessment which includes: i) informed consent process through RedCap; ii) 6 minute walk test (6MWT) using a standardized protocol; iii) self-report questionnaires; iv) instructions on ActiGraph wear with special attention to explaining the importance of adherence and reducing reactivity (e.g., ensuring that participants know not to make changes in activity levels during the week long baseline assessment, and developing individualized plans for ActiGraph wear including option for Twilio text reminders). Participants will next be randomized to one of the 2 conditions. Allocation will be performed by each site study coordinator using REDCap. Each program involves audio-recorded group sessions for 10 consecutive weeks, 60 minutes per week at a time that is convenient to participants. Participants will receive a check-in message via email or mail from the study team in between post-tests and follow-up assessments to maintain study relationships. All participants will be offered the other group's content (i.e., group manual and website) at the end of the 6-month follow up assessments.

All participants will wear the ActiGraph devices at the baseline, post-intervention, and 6-month follow up assessments. All participants will be instructed on ActiGraph maintenance using standard operating procedures used in a prior feasibility trial at MGH. Participants will return the ActiGraph watches after the assessments, provided they have logged seven consecutive days of valid data. Participants with missing data will be instructed to continue wearing the device until seven days have been reached. The RA will provide participants with ActiGraphs either in-person or via mail, and participants will be asked to return their devices during in-person assessments, during sessions, or via mail with a prepaid postage envelope. The RA will remotely monitor ActiGraph adherence using CentrePoint Sync. For participants in the *GetActive-Fitbit* condition, participants will be asked to wear a Fitbit for the duration of the program and sync the Fitbit to a Fitbit application on their smartphone. Using the Fitbit steps, the RA will determine a new step count goal weekly based on whether the *GetActive-Fitbit* participant met their step goal in the prior week. If a participant met their step-count goal, they will increase their steps by 10%. If they did not meet the goal, they will continue with the same goal. If they do not meet the goal for 2 consecutive weeks, their goal will be decreased by 10%.

Aim 3 will commence following Aim 2, and this aim of the study is intended to optimize fidelity and study implementation protocol procedures in preparation for a future fully powered multisite efficacy RCT. This aim will entail conducting focus groups with staff and patients to refine study materials and procedures. A future IRB submission will detail this aim in greater detail.

4. Subject Selection

An important goal of this proposal is to improve upon our R34 trial at MGH and recruit a diverse sample that is representative of the USA demographics with regard to race and ethnicity. Our goal is to follow the race and ethnicity distribution of the USA (below) and recruit at least 38% racial and ethnic minorities. The MGH Pain Clinic population includes ~80% Whites/Caucasians, and this was reflected in the sample we obtained with our R34 pilot study. Specifically, within our feasibility RCT at MGH our sample was primarily White (80%) and Not Hispanic (85%). The enrolled sample included 3% American Indian/Alaskan, 4% Asian, 9% Black/African American and 4% Multi-race. During this R01 we will directly focus on recruitment of racial and ethnic minorities at all sites including MGH. Yearly at MGH, we will have available 95 American Indians/Alaskan, 127 Asians, 286 Blacks/African Americans, and 127 individuals who identify as Multiracial. The Rush Pain Clinic treats chronic pain patients who are diverse with regard to race/ethnicity and sex. In our recent R01 NR013910 our sample corresponded with regard to race and ethnicity to the general sample seen at our pain clinic. Across the entire sample 51% were Black/African American, 35% white, 7% multiracial and 6% other race. With regard to ethnicity 10% were Hispanic/Latino and 90% Non-Hispanic/Latino. The Duke Pain Clinics in Durham, NC serve patients primarily that live in Durham, NC as well as the larger full count of Durham and outlying rural areas. Durham county 2020 reports the population to be 48% White, 40 African-American, 5% Asian, and 7% other races. In an ongoing R01 recruiting patients with persistent pain at Duke University Medical Center, of 291 recruited participants race is 60% White, 34% African-American, 3% Asian, and 5% other. In this work, we will be able to increase our recruitment numbers to match with the county wide statistics and recruit participants that are no more than 48% White and no less than 40% African-American, 5% Asian, and 7% another race.

We developed strategies to ensure recruitment of racial and ethnic minority samples, including: 1) encouraging referring physicians and all study staff to engage in trainings around cultural competence and internalized racism to ensure interactions with patients are able to be rooted in trust and respect; 2) clearly explaining the research study and ensure that patients have all information needed to make a decision regarding participation, to helps to address mistrust of research and underline respect for patient

autonomy; 3) at MGH, we will approach all racial and ethnic minorities. This will not be done at Rush and Duke given that their racial and ethnic representation is much more diverse. The goal will be to ensure that patients of all racial and ethnic backgrounds feel that they are part of a community that aims to improve quality of care for chronic pain patients.

Inclusion/Exclusion Criteria

Inclusion Criteria

1. Male and female outpatients, age 18 years or older
2. Fluent in English
3. Have nonmalignant chronic musculoskeletal pain for more than 3 months
4. Able to perform a 6-minute walk test (6MWT)
5. Owns a smartphone with Bluetooth 4.0 or computer
6. Willingness and ability to participate in one of the 2 programs and to comply with the requirements of the study protocol.
7. Free of concurrent psychotropic medication for at least 2 weeks prior to initiation of treatment, OR stable on current medication for a minimum of 6 weeks and willing to maintain a stable dose
8. Low-level of activity (must meet 2/3 criteria; if difficult to assess, recruiter may ask participant to report their recent step data from their smartphone to better understand their activity level)
    a. Sitting for more than 8 hours/day per self-report
    b. Exercising for fewer than 3 days/week (30+min of exercise constitutes an "exercise day") per self-report
    c. Walking for fewer than 7 days/week (30+min of walking constitutes a "walking day") per self-report

Exclusion Criteria

1. Diagnosed with a medical illness expected to worsen in the next 6 months (e.g., malignancy)
2. Serious mental illness or instability for which hospitalization may be likely in the next 6 months
3. Current active suicidal ideation with plan or intent reported on self-report
4. Untreated and/or unstable schizophrenia, bipolar disorder, or other psychotic disorder
5. Current, active substance abuse or dependence, and current alcohol dependence within the past 6 months
6. Practice of yoga, meditation, guided imagery or other mind body techniques that elicit the RR, once per week for >45 min 3 times/week within the last 3 months or less.
7. Regular use of Fitbit DMD in the last 3 months
8. Unable to walk/in a wheelchair
9. Mild cognitive decline for participants 65+ only (Short Portable Mental Status Questionnaire; PMSQ 4 or more errors).

Participants will be adult patients with chronic, heterogeneous, non-malignant pain diagnosed by their medical provider. Participants will only include English speakers, due to study resources and access to translators. Participants who do not have access to a personal smartphone equipped with Bluetooth 4.0 or later may be provided with one. As in our feasibility R34 we anticipate that the majority of recruitment will occur from the Pain Clinics affiliated with each site, due to an overabundance of available study participants and due to the larger goal of implementation of programs within the Pain Clinic. Adults with

chronic pain will be recruited from physician referrals and IRB approved flyers throughout all MGH, Duke, and Rush outpatient clinics and community health centers, social media, and from the community. In the event that a provider refers a patient without sharing contact information and the patient agrees to be contacted by the study team, the research assistant will use the patient's medical record to access this information. Participants may also be recruited via referrals from related research studies, if they agree to be contact by our study team. In addition to the shared recruitment strategies, each site will have site-specific methods of recruitment. MGH will utilize the Mass General Brigham Rally platform to recruit patients from the greater Boston area. Duke University will also recruit via medical record review. Rush will also promote the study during the hospital "hold message" when people call Rush Medical Center. Screening will take place remotely and consent will occur in person.

5. Subject Enrollment

The screening process will take place remotely and consent will occur in person. Remote screening will be conducted by the research assistant over the phone. During the screening call, a research assistant will provide study details to participants and assess eligibility via self-report questions. Participants will also have the option of completing a portion of this screening on their own via REDCap. There will be a QR code and link directing potential participants to the screening REDCap on our study flyers. If eligible after individual screening, the participant will receive a call from the research assistant. A research assistant will briefly review the consent form with eligible participants who express interest and intent to participate in the study. The research assistant will ask eligible participants to provide (1) their height and weight information, to initialize their ActiGraph, (2) dates and times that they are available to attend the group program and maintain a detailed log of this information. Study staff will continue to follow-up with interested participants via phone until a date for the groups has been selected (group times are based off a majority of participants' availability). Once a mutual date and time for the groups has been selected, a research assistant will call eligible participants to schedule an in-person group baseline assessment at each of the 3 sites, which includes the informed consent procedure. The research assistant will ask eligible participants who are not available at the set time if they want to be contacted for the next round of groups or for future trials. Though the timeline to hear back from the study team may vary, participants on the waiting list can expect to hear from us within a month and are guaranteed to be recontacted. Individuals who do not meet study criteria will be offered a resource sheet with relevant health and mental health information. During the in-person baseline assessment, participants will sign consent forms electronically on REDCap through the eConsent framework.

6. Study Procedures

Eligible participants who are able to attend the scheduled group sessions will meet in person at each of the 3 sites for the baseline assessment. If other eligible participants are available at the same time, there is an option to conduct the baseline concurrently as a group. The baseline assessment includes: i) informed consent process through REDCap; ii) 6 minute walk test (6MWT) using a standardized protocol; iii) self-report questionnaires; iv) instructions on ActiGraph wear with special attention to explaining the importance of adherence and reducing reactivity (e.g., ensuring that participants know not to make changes in activity levels during the week long baseline assessment, and developing individualized plans for ActiGraph wear including option for Twilio text reminders). Participants will next be randomized to one of the 2 conditions. Allocation will be performed by each site study coordinator using REDCap. Each program involves audio-recorded group sessions (e.g., ~4-10 participants per group) for 10 consecutive weeks, 60 minutes per week at a time that is convenient to participants. Program sessions will be led by clinical psychologists who have undergone extensive training in these interventions and have expertise in

chronic pain. Participants will also be invited to participate in a group exit interview following a semi-structured script. The group interview will occur during the post-intervention assessment. Those who are interested in participating but cannot attend the group interview session can participate individually at another time. All participants will also be given the opportunity to share contact information after the post-test assessments to maintain the community and relationships developed during the in-person group sessions.

The *GetActive-Fitbit* program is a group mind-body program developed iteratively from the Relaxation Response Resiliency Program (3RP) that incorporates additional cognitive behavioral and physical restoration skills (e.g., quota-based pacing) to help individuals increase self-reported, performance-based, and objectively measured (step count) physical function. Participants randomly assigned to this intervention condition will receive daily text/email reminders to practice program skills, record their walking, and record their skills practice via methods such as Twilio, if they consent to receive text reminders. A health education program ("Healthy Living for Pain") will serve as the comparison condition for this aim of the study and will teach participants about health topics such as sleep, nutrition, and exercise. Participants randomly assigned to this comparison condition will receive journaling homework with daily text/email reminders. Participants will be given the option to share a copy of their journal with the study clinician throughout the program.

All participants will wear the ActiGraph wGT3X-BT devices to measure step-count at baseline, post-intervention, and 6-month follow-up assessments. All participants will be instructed on ActiGraph maintenance using standard operating procedures used in a prior feasibility trial at MGH. Participants will return the ActiGraph watches after the assessments, provided they have logged seven consecutive days of valid data. Participants with missing data will be instructed to continue wearing the device until seven days have been reached. The RA will provide participants with ActiGraphs either in-person or via mail, and participants will be asked to return their devices during in-person assessments, during sessions, or via mail with a prepaid postage envelope. The RA will remotely monitor ActiGraph adherence using CentrePoint Sync. For participants in the *GetActive-Fitbit* condition, participants will be asked to wear a Fitbit Inspire 3 for the duration of the program and sync the Fitbit to a Fitbit application on their smartphone. Using the Fitbit steps, the RA will determine a new step count goal weekly based on whether the *GetActive-Fitbit* participant met their step goal in the prior week. If a participant met their step-count goal, they will increase their steps by 10%. If they did not meet the goal, they will continue with the same goal. If they do not meet the goal for 2 consecutive weeks, their goal will be increased by 10% of the average step count they had accomplished the week prior. Study clinicians may also conduct independent check-ins with participants who do not meet their goal to ensure barriers are known. The standard baseline goal for all participants randomized to the *GetActive-Fitbit* condition is that they experience a 1,000 step increase by the end of the program, though this will be variable depending on participant ability.

The following measures will be administered at all timepoints, to participants in both study arms, unless otherwise stated. The measures assess physical and emotional function, behavior, medical symptoms, and coping strategies with pain. These measures will be administered on hospital-owned encrypted devices or paper and pencil, depending on participant comfort, and should take about 30 minutes to complete.

Demographics (baseline only)
Treatments (post-tests and follow-up only)
Pain, Enjoyment of Life and General Activity scale (PEG-3)
WHO Disability Assessment Scale (WHODAS)

6 Minute Walk Test (6MWT)
ActiGraph wGT3X-BT Accelerometer
PROMIS depression v1.08b
PROMIS anxiety v1.08a
Pain Catastrophizing Scale (PCS)
Tampa Kinesiophobia Scale (TSK)
Measure of Current Status (MOCS)
Cognitive and Affective Mindfulness Scale (CAMS)
Modified Global Impression of Change (MGIC) – only at post-test
Credibility/Expectancy Questionnaire (CEQ) (post-tests or as soon as possible after post-tests)
Client Satisfaction Questionnaire (CSQ-3) (post-tests or as soon as possible after post-tests)
Fitbit Use (GetActive Only)- only at 6-month follow-up

Table 1. Study Procedures

Time	Task	Details
6-7 Weeks Before Intervention	Recruitment and Screening	RA receives referrals, screens via telephone, records availability for eligible participants
3 Weeks Before Intervention	Scheduling	RA selects baseline time based on participant availability. RA calls participants to confirm time.
~1 Week Before Intervention	Consent and Baseline Assessment	Review and sign consent. Participants complete: self-report measures, 6-minute walk test, Participants also receive ActiGraph devices and instructions.
10-Week Intervention	Group Sessions 1-10	Participants complete 10 weeks of group sessions of either *GetActive-Fitbit* or *Healthy Living for Pain.*<br><br>Sessions will be audio recorded, to ensure accuracy of delivery and assess fidelity.
1 Week Post Intervention	Post-Intervention Assessments	Participants repeat procedures from the baseline assessment. Participants return ActiGraph devices after the post-test assessment in person.
2 Weeks Before 6-Month Assessments	ActiGraphs Mailed Out	RA mails ActiGraphs to participants to be worn in week prior to 6-Month Follow up
6 Months Post Intervention	6-Month Follow-Up Assessments	Participants repeat procedures from the baseline and post-test assessments. Participants return ActiGraph devices after the 6-month follow-up assessment in person.

Participants will be paid $945 for completing the study in its totality. If participants do not complete the study, we will pay based on the following study visits and tasks completed:

- For the baseline assessment, participants will receive $60 for the completion of study tasks and up to $100 additional dollars for ActiGraph wear ($10 dollar per day, plus an additional $30 for 7 days with complete valid wear).
- For the post-test assessment, participants will receive $100 for the completion of study tasks and up to $155 additional dollars for ActiGraph wear ($15 dollar per day, plus an additional $50 for 7 days with complete valid wear).
- For the 6-month follow-up assessment, participants will receive $100 for the completion of study tasks and up to $190 additional dollars for ActiGraph wear ($20 dollar per day, plus an additional $50 for 7 days).
- For transportation costs for session attendance, participants will be paid $150 for travel compensation ($15 per session) and can receive an additional $50 if 8/10 sessions are attended.
- For the optional exit interview, participants will receive $40.

Participants should expect to receive payment following each major assessment timepoint (i.e., baseline, post-test, and 6-month follow-up) as well as following the program sessions. MGH and Rush will pay participants via checks, as per their respective hospitals payment systems, and Duke will pay participants via ClinCards, per their hospital system.

7. Risks and Discomforts

Patients will be informed that the foreseeable physical risks from this research study are minimal. They will be informed that there is some risk of breach of privacy/confidentiality associated with the use of written communication (e.g., emailing, text messages) and audio recording software. They will also be informed of the unlikely situation that they might feel uncomfortable with the topics discussed within the group, and they can alert the group leader who will provide help, as needed. The group leaders are experienced clinical psychologists who have specific expertise with chronic pain in adults. They will also be informed that they may feel uncomfortable completing various psychological questionnaires and that they may find it time-consuming to participate in weekly 60-minute groups.

In the unlikely event that a participant is determined to be in distress or actively suicidal and at risk for self-harm during any study procedure, we will use a standardized protocol for assessing and monitoring risk developed by Dr. Vranceanu that has been successfully used in other remote trials. In this protocol, the research assistant would contact the MPI (Vranceanu) and the appropriate clinical intervention would be executed. In case we are unable to contact the participant we will contact their safety contacts (see Potential Risks to participant section). In case suicidality is determined during the intervention or control sessions, the study clinicians will perform safety procedures in real time and immediately contact the MPIs. Further, we set serious mental illness and current suicidal ideation as exclusion criteria to further reduce the chance that enrolled participants would express harm towards themselves or others at any point during the study.

Direct adverse events from increasing walking are not expected. The increase in activity will be safe, quota-based and gradual, contingent upon meeting step count criteria in the previous week. All study staff have been trained in responsible conduct of research. Adverse events during the study will be tracked as part of the daily adherence/home practice log. Specifically, participants will be asked if they experienced any negative outcomes because of becoming more physically active during that day. Any participants reporting adverse events will be immediately contacted and, if necessary, assessed by the study clinician before being referred for appropriate care as needed.

As mentioned above, we will be using secure email to communicate with participants. All email communication with participants will be encrypted using the send secure function. To further protect participants' confidentiality, we will discourage participants from communicating about medical issues by non-secure email.

8. Benefits

We expect many benefits from study participations among individuals in *GetActive-Fitbit* including improvement in physical (self-report, performance and objective) and emotional (depression and anxiety) function, increased coping ability, and decreases catastrophizing, fear of pain, and pain intensity. All participants are expected to benefit from the support of the therapist and group members. The potential benefits from this study far outweigh the potential risks. The data collected as part of this study may ultimately help researchers and clinicians better care for patients with chronic pain.

9. Statistical Analysis

We plan to enroll N=120 participants across sites, with 40 participants recruited per site. We believe that this sample size is appropriate for a feasibility trial, including similar proposals previously funded NCCIH (e.g., 1U01AT010462). The proposed sample provides good power to achieve our multisite fidelity and feasibility benchmarks. Specifically, if 90 participants are enrolled, the study will have an 80% probability of meeting all seven Aim 2 feasibility benchmarks if the expected proportion of participants enrolling, completing at least 7 of 10 sessions, rating the program as credible and satisfactory, and completing post-treatment follow-up is at least 89% and if the expected proportion of protocol checklists deemed accurate is at least 95%. Moreover, the planned sample size will provide adequate precision for estimating fidelity and feasibility benchmarks.

Statistical analyses:
First, we will report:

- percent eligible sedentary chronic pain patients who enroll after being determined eligible during screening (benchmark ≥ 80% AND 34/90 racial and ethnic minorities).
- Total duration of recruitment in months overall and at each site.
- Percent of patients who attend ≥7/10 sessions for each program (benchmark is 80%).
- Percent patients with credibility scores higher than the scale's mid point (Credibility and Expectancy scale; benchmark is 80%)
- Percent patients with satisfaction scores higher than the scale's mid point (Client Satisfaction scale; benchmark is 80%)
- Percent patients who complete post-treatment (benchmark is 80%) and 6 months follow up (benchmark is 70%) including self-report, performance based and Actigraph valid data.
- Percent accuracy of standardized protocol checklists for all assessments (benchmark is 90% accuracy).
- We will also report additional information necessary to complete the CONSORT diagram.

Next, we will use Fisher's exact tests to make comparisons of number of people meeting each benchmark across sites and between the intervention arms, while also plotting performance metrics across sites to account and assess for trends in differences due to the low statistical power.

Estimates of variance components required for design of a future efficacy trial will be obtained from preliminary analysis of outcome report, performance based, and objective/ambulatory measurements in mixed model repeated-measures analyses.

10. Monitoring and Quality Assurance

The MPIs will monitor the validity of the data and adherence to the IRB approved protocol on a daily basis. A member of study staff will verify that all items on all questionnaires have been addressed. Data will be checked for out of range values using frequency distributions prior to analyzing the data. The MPIs will be responsible for ensuring compliance with IRB procedures.

- *Ongoing monitoring*: For each intervention session, an intervention checklist will be completed, detailing the information and exercises covered per session. Study therapists will undergo weekly supervision. All intervention sessions will be audio-recorded.

Participant Confidentiality. All study materials will identify participants only by study ID, visit number, and visit date. The study research assistant will be trained in procedures to protect study participant confidentiality. Recordings of each session will be immediately de-identified (e.g., only ID, visit number and date) and stored on a password protected computer; the recording will be subsequently and immediately deleted from the recording device.

Data Validity and Security. The research team will develop standard operating procedures for data collection, data management, and quality control. Drs. Vranceanu and Greenberg will oversee all procedures related to secure data collection and management, along with Dr. Parker and the project manager. Electronic information will be stored in REDCap (Research Electronic Data Capture), a free, secure, and HIPAA-compliant web-based application hosted by the Partners HealthCare Research Computing Enterprise Research Infrastructure & Services (ERIS) group (based at the PHS Needham corporate datacenter). All data will be stored on password protected computers that will be stored in secure locations at all times. Only research staff will have access to these data locations.

Participants' data will be identified by an ID number only, and a link between names and ID numbers will be kept on a separate folder on a Partners DropBox. Recordings will be immediately deleted from the digital recording device and stored under password protection. Among the 3 recruitment sites (MGH, Duke, Rush), PHI will be shared related to enrolled study participants. MGH will send and receive PHI to/from Duke and Rush using encrypted email as well as shared storage in the Partners-approved platform Dropbox. This PHI will be shared as sparingly as possible, but may include sharing names, ages, and contact information, among other identifiers. PHI will not be shared with any study collaborators outside of MGH, Duke, and Rush.

Data from this study will be stored for seven years after the publication of all study results, at which time all paper screening and consent forms will be shredded, and computer files will be deleted.

10.1 Review and Report Adverse Events and Unanticipated Problems

If the research team identifies an adverse event (e.g., via study participants, research team members, clinicians, or review of electronic health records), we will follow the steps below:

Serious Adverse Events. Any event that meets the FDA definition of a serious adverse event or that is deemed by the research team or safety and monitoring committee to impose significant hazard, contraindication, side effect, or precaution, will involve expedited review. All relevant information will be reported to the committee via secure e-mail within 2 days. The committee will review the information and determine the extent to which the event was relevant to the study procedures. Reporting to the IRB will be done within 24 hours; reporting to NIA (including responses of the committee and the IRB) will be done within 7 days.

Non-Serious Adverse Events. Per study year and at study completion, the data safety and monitoring committee will receive a summary of adverse events per randomized group, including number and types of events, severity, time point, and determination of whether the events were study related.

Study Stopping Rules. Give low risk associated with this behavioral study, we have not identified a stopping rule. However, during the study period, if the data safety and monitoring committee deems that participant risk outweighs participant benefit, the committee may choose to recommend study termination.

10.2 External Factors That May Impact Participant Safety or Study Ethics

Dr. Vranceanu and Greenberg will review relevant literature at least twice per study year to ensure that no new developments may affect the study or participants. The study will not place limits on usual care and all activities will be conducted over and above what patients and caregivers normally receive as part of usual care.

11. Select the Privacy and Confidentiality measures that apply to this research:

- ☒ Study procedures will be conducted in a private setting
- ☒ Only data and/or specimens necessary for the conduct of the study will be collected
- ☒ Data collected (paper and/or electronic) will be maintained in a secure location with appropriate protections such as password protection, encryption, physical security measures (locked files/areas)
- ☒ Specimens collected will be maintained in a secure location with appropriate protections (e.g. locked storage spaces, laboratory areas)
- ☒ Data and specimens will only be shared with individuals who are members of the IRB-approved research team or approved for sharing as described in this IRB protocol
- ☒ Data and/or specimens requiring transportation from one location or electronic space to another will be transported only in a secure manner (e.g. encrypted files, password protection, using chain-of-custody procedures, etc.)
- ☒ All electronic communication with participants will comply with Mass General Brigham secure communication policies
- ☒ Identifiers will be coded or removed as soon as feasible and access to files linking identifiers with coded data or specimens will be limited to the minimal necessary members of the research team required to conduct the research
- ☒ All staff are trained on and will follow the Mass General Brigham policies and procedures for maintaining appropriate confidentiality of research data and specimens
- ☒ The PI will ensure that all staff implement and follow any Research Information Service Office (RISO) requirements for this research
- ☐ Additional privacy and/or confidentiality protections

Describe below:

12. References

1. Coronado, V. G. *et al.* Surveillance for traumatic brain injury-related deaths--United States, 1997-2007. *MMWR. Surveill. Summ.* **60**, 1–32 (2011).
2. Ponsford, J. *et al.* Factors influencing outcome following mild traumatic brain injury in adults. *J. Int. Neuropsychol. Soc.* **6**, 568–579 (2000).
3. Boake, C. *et al.* Diagnostic Criteria for Postconcussional Syndrome After Mild to Moderate

Traumatic Brain Injury. *J. Neuropsychiatry Clin. Neurosci.* **17**, 350–356 (2005).
4. Silverberg, N. D. *et al.* Systematic Review of Multivariable Prognostic Models for Mild Traumatic Brain Injury. *J. Neurotrauma* **32**, 517–526 (2015).
5. Moore, E. L., Terryberry-Spohr, L. & Hope, D. A. Mild Traumatic Brain Injury and Anxiety Sequelae : A Review of the Literature Mild Traumatic Brain Injury and Anxiety Sequelae : A Review of the Literature. *Brain Inj.* **20**, 117–132 (2006).
6. Scholten, A. C. *et al.* Prevalence of and Risk Factors for Anxiety and Depressive Disorders after Traumatic Brain Injury: A Systematic Review. *J. Neurotrauma* **33**, 1969–1994 (2016).
7. Zahniser, E. *et al.* The Temporal Relationship of Mental Health Problems and Functional Limitations Following mTBI: A TRACK-TBI Study. *J. Neurotrauma* **Dec 13**, (2018).
8. Broshek, D. K., De Marco, A. P. & Freeman, J. R. A review of post-concussion syndrome and psychological factors associated with concussion. *Brain Injury* **29**, 228–237 (2015).
9. Pennebaker, J. W. Psychological factors influencing the reporting of physical symptoms. *The Science of Self-Report: Implications for research and practice* 299–315 (2000).
10. Fortney, S. M., Schneider, V. S. & Greenleaf, J. E. The physiology of bed rest. *Compr. Physiol.* 889–939 (2011). doi:https://doi.org/10.1002/cphy.cp040239
11. Silverberg, N. D. & Iverson, G. L. Is rest after concussion 'the best medicine?': Recommendations for activity resumption following concussion in athletes, civilians, and military service members. *J. Head Trauma Rehabil.* **28**, 250–259 (2013).
12. Tüzün, E. H. Quality of life in chronic musculoskeletal pain. *Best Pract. Res. Clin. Rheumatol.* **21**, 567–579 (2007).
13. Zale, E. L., Lange, K. L., Fields, S. A. & Ditre, J. W. The relation between pain-related fear and disability: A meta-analysis. *Journal of Pain* **14**, 1019–1030 (2013).
14. Greenberg, J. *et al.* Pain catastrophizing and limiting behavior mediate the association between anxiety and post-concussion symptoms. *Psychosomatics* (2019). doi:https://doi.org/10.1016/j.psym.2019.09.004
15. Astin, J. A., Shapiro, S. L., Eisenberg, D. M. & Forys, K. L. Mind-Body Medicine: State of the Science, Implications for Practice. *J. Am. Board Fam. Med.* **16**, 131–147 (2003).
16. Wahbeh, H., Elsas, S. M. & Oken, B. S. Mind-body interventions: Applications in neurology. *Neurology* **70**, 2321–2328 (2008).
17. Hofmann, S. G., Sawyer, A. T., Witt, A. A. & Oh, D. The Effect of Mindfulness-Based Therapy on Anxiety and Depression: A Meta-Analytic Review. *J. Consult. Clin. Psychol.* **78**, 169–183 (2010).
18. Ungar, T. & Knaak, S. Mental health stigma and the mind-body problem: Making the case for strategic collusion. *Australian and New Zealand Journal of Psychiatry* **47**, 1088 (2013).
19. Erwin Wells, R., Phillips, R. S. & McCarthy, E. P. Patterns of mind-body therapies in adults with common neurological conditions. *Neuroepidemiology* **36**, 46–51 (2011).
20. Rose, R. D. *et al.* A randomized controlled trial of a self-guided, multimedia, stress management and resilience training program. *Behav. Res. Ther.* **51**, 106–112 (2013).
21. Greenberg, J. *et al.* Reduced interference in working memory following mindfulness training is associated with increases in hippocampal volume. *Brain Imaging and Behavior* 1–11 (2018). doi:10.1007/s11682-018-9858-4
22. Glueckauf, R. L. & Ketterson, T. U. Telehealth interventions for individuals with chronic illness: Research review and implications for practice. *Professional Psychology: Research and Practice* **35**, 615–627 (2004).
23. Vranceanu, A. M. *et al.* Mind-body therapy via videoconferencing in patients with neurofibromatosis: An RCT. *Neurology* **87**, 806–814 (2016).
24. Vranceanu, A. M. *et al.* Mind-Body Program Delivered via Live Videoconferencing to International English-Speaking Adults with Neurofibromatosis: Study Protocol for a Single-Blind Randomized Controlled Trial (Preprint). *JMIR Res. Protoc.* **7**, (2018).

25. Vranceanu, A. M. *et al.* Results of a feasibility randomized controlled trial (RCT) of the Toolkit for Optimal Recovery (TOR): a live video program to prevent chronic pain in at-risk adults with orthopedic injuries. *Pilot Feasibility Stud.* **5**, (2019).
26. Kelly, J. P. Concussion in Sports and Recreation. *Semin. Neurol.* **20**, 165–172 (2000).
27. Harmon, K. G. Assessment and management of concussion in sports. *Am. Fam. Physician* **60**, 887–894 (1999).
28. Bruns, J. & Hauser, W. A. The Epidemiology of Traumatic Brain Injury: A Review. *Epilepsia* **44**, 2–10 (2003).
29. Kaut, K. P., DePompei, R., Kerr, J. & Congeni, J. Reports of head injury and symptom knowledge among college athletes: Implications for assessment and educational intervention. *Clin. J. Sport Med.* (2003). doi:10.1097/00042752-200307000-00004
30. NIH Consensus Development Panel on Rehabilitation of Persons with Traumatic Brain Injury. *Jama* **282**, 974–983 (1999).
31. Beiter, R. *et al.* The prevalence and correlates of depression, anxiety, and stress in a sample of college students. *J. Affect. Disord.* **173**, 90–96 (2015).
32. Pujol, J., Vendrell, P., Junqué, C., Martí-Vilalta, J. L. & Capdevila, A. When does human brain development end? Evidence of corpus callosum growth up to adulthood. *Ann. Neurol.* **34**, 71–75 (1993).
33. Lungu, A. & Sun, M. Time for a Change: College Students' Preference for Technology-Mediated Versus Face-to-Face Help for Emotional Distress. *Telemed. e-Health* **22**, 991–1000 (2016).
34. Plummer, F., Manea, L., Trepel, D. & McMillan, D. Screening for anxiety disorders with the GAD-7 and GAD-2: A systematic review and diagnostic metaanalysis. *Gen. Hosp. Psychiatry* **39**, 24–31 (2016).
35. Harris, P. A., Taylor, R., Thielke, R., Payne, J., Gonzalez, N., & Conde, J. G. Research electronic data capture (REDCap)--a metadata-driven methodology and workflow process for providing translational research informatics support. *Journal of biomedical informatics*, 42, 377–381. (2009).
