# Supplementary material for: Feasibility of a Mind-Body Program for Chronic Pain: A Randomized Clinical Trial
Source: JAMA Netw Open. 2025 Jun 16;8(6):e2515685. doi: 10.1001/jamanetworkopen.2025.15685 (PMC12171935; doi:10.1001/jamanetworkopen.2025.15685)
Supplement: Supplement 3. — Data Sharing Statement [file jamanetwopen-e2515685-s003.pdf]

Data Sharing Statement

Greenberg. Feasibility of a Mind-Body Program for Chronic Pain. *JAMA Netw Open*. Published June 16, 2025. doi:10.1001/jamanetworkopen.2025.15685

Data
Additional Information: clinicaltrials.gov; NCT05700383
Data available: Yes
Data types: Deidentified participant data
How to access data: Data can be made available upon reasonable request by emailing avranceanu@mgh.harvard.edu
When available: With publication

Supporting Documents
Document types: None

Additional Information
Who can access the data: anyone requesting the data
Types of analyses: for any purpose
Mechanisms of data availability: data can be made available upon request from corresponding author
